# Catestatin in innate immunity and Cateslytin-derived peptides against superbugs

**DOI:** 10.1038/s41598-021-94749-6

**Published:** 2021-08-02

**Authors:** Francesco Scavello, Angela Mutschler, Sophie Hellé, Francis Schneider, Sylvette Chasserot-Golaz, Jean-Marc Strub, Sarah Cianferani, Youssef Haikel, Marie-Hélène Metz-Boutigue

**Affiliations:** 1 BioMaterials and BioEngeneering, Institut National de la Santé et de la Recherche Médicale UMR_S 1121, Federation of Translational Medicine Faculty, of Odontology, University of Strasbourg, Hôpital Civil, Porte de L’Hôpital, 67000 Strasbourg, France; 2 Department of Biology, Ecology and Earth Science, University of Calabria, Arcavacata di Rende, Italy; 3 Faculty of Medicine, University of Strasbourg, Strasbourg, France; 4 Médecine Intensive-Réanimation, Hautepierre Hospital, Hôpitaux Universitaires, Strasbourg, Federation of Translational Medicine, Faculty of Medicine, University of Strasbourg, Strasbourg, France; 5 Centre National de la Recherche Scientifique, Institut des Neurosciences Cellulaires et Intégratives, University of Strasbourg, Strasbourg, France; 6 Centre National de la Recherche Scientifique, Laboratory of Bio-Organic Mass Spectrometry, Analytical Sciences Department, Pluridisciplinary Institute Hubert Curien, UMR 7178, University of Strasbourg, Strasbourg, France; 7 Faculty of Odontology, University of Strasbourg, Strasbourg, France

**Keywords:** Biochemistry, Microbiology, Diseases, Endocrinology

## Abstract

Chromogranin A (CgA) is the precursor of several antimicrobial peptides, such as Catestatin (Cts, bovine CgA344-364), initially described as a potent inhibitor of catecholamines. This peptide displays direct antimicrobial activities and contributes to immune system regulation. The aim of the present study is to investigate a designed peptide based on Cts to fight infections against superbugs and more particularly *Staphylococcus aureus*. In addition to Cateslytin (Ctl, bovine CgA344-358), the active domain of Catestatin, several peptides including dimers, D-isomer and the new designed peptide DOPA-K-DOPA-K-DOPA-TLRGGE-RSMRLSFRARGYGFR (Dopa_5_T-Ctl) were prepared and tested. Cateslytin is resistant to bacterial degradation and does not induce bacterial resistance. The interaction of Catestatin with immune dermal cells (dendritic cells DC1a, dermal macrophages CD14 and macrophages) was analyzed by using confocal microscopy and cytokine release assay. The dimers and D-isomer of Ctl were tested against a large variety of bacteria showing the potent antibacterial activity of the D-isomer. The peptide Dopa_5_T-Ctl is able to induce the self-killing of *S. aureus* after release of Ctl by the endoprotease Glu-C produced by this pathogen. It permits localized on-demand delivery of the antimicrobial drug directly at the infectious site.

## Introduction

Some infections caused by antimicrobial-resistant microorganisms often no longer respond to conventional antibiotics^1^. In the last decade, several multidrug-resistant high-risk strains have evolved due to the selective pressure of antimicrobial use^2^. The ever increasing number of such microorganisms has become a great and global public health threat worldwide among vulnerable populations such as immuno-suppressed and critically ill patients^3^. There is an urgent need to develop new antimicrobial compounds to fight against these superbugs. Host-defense peptides (HDPs) have emerged as new attractive candidates in the development of new antimicrobial agents^4^. Recently, a growing body of evidence suggests the biology and immunology of HDPs extends beyond the classical direct antimicrobial activity with the regulation of immune cells properties^5^.

We have previously shown that the pro-hormone chromogranin A (CgA) is the precursor of several physiological antimicrobial peptides^6–9^. In particular, CgA is an acidic glyco-phospho-protein stored in secretory vesicles of numerous nervous, neuroendocrine and immune cells. It is proteolytically cleaved by prohormone convertases, cathepsin L, plasmin and kallikrein to generate biologically active peptides released upon stress in most of the body fluids^10^. One of these HDPs, Catestatin (Cts, bovine CgA344-364) was initially described as a potent inhibitor of catecholamines release^11^. In addition, Cts plays a crucial role in cardiovascular system^12,13^, it displays direct antimicrobial activities^9^, it contributes to the immune system regulation and it modulates severe inflammatory response^14–20^. It was established that Cateslytin (Ctl, bovine CgA344-358) is the active core of Cts^9,21^ and it is resistant to degradation by the proteases produced by *Staphylococcus aureus*^22^. Cts and Ctl show a broad spectrum of antimicrobial activities against bacteria and yeasts once attached to biomaterials^23,24^. A new strategy has been proposed by linking Cts to materials through a spacer which can be cleaved by hyaluronidase, an enzyme from *S. aureus* and *Candida albicans*^23^. Our group has previously characterized the antimicrobial and mechanistic properties of the D-isomer of Ctl, where all L-amino acids were substituted by D-amino acids^25,26^.

**Figure 1:**
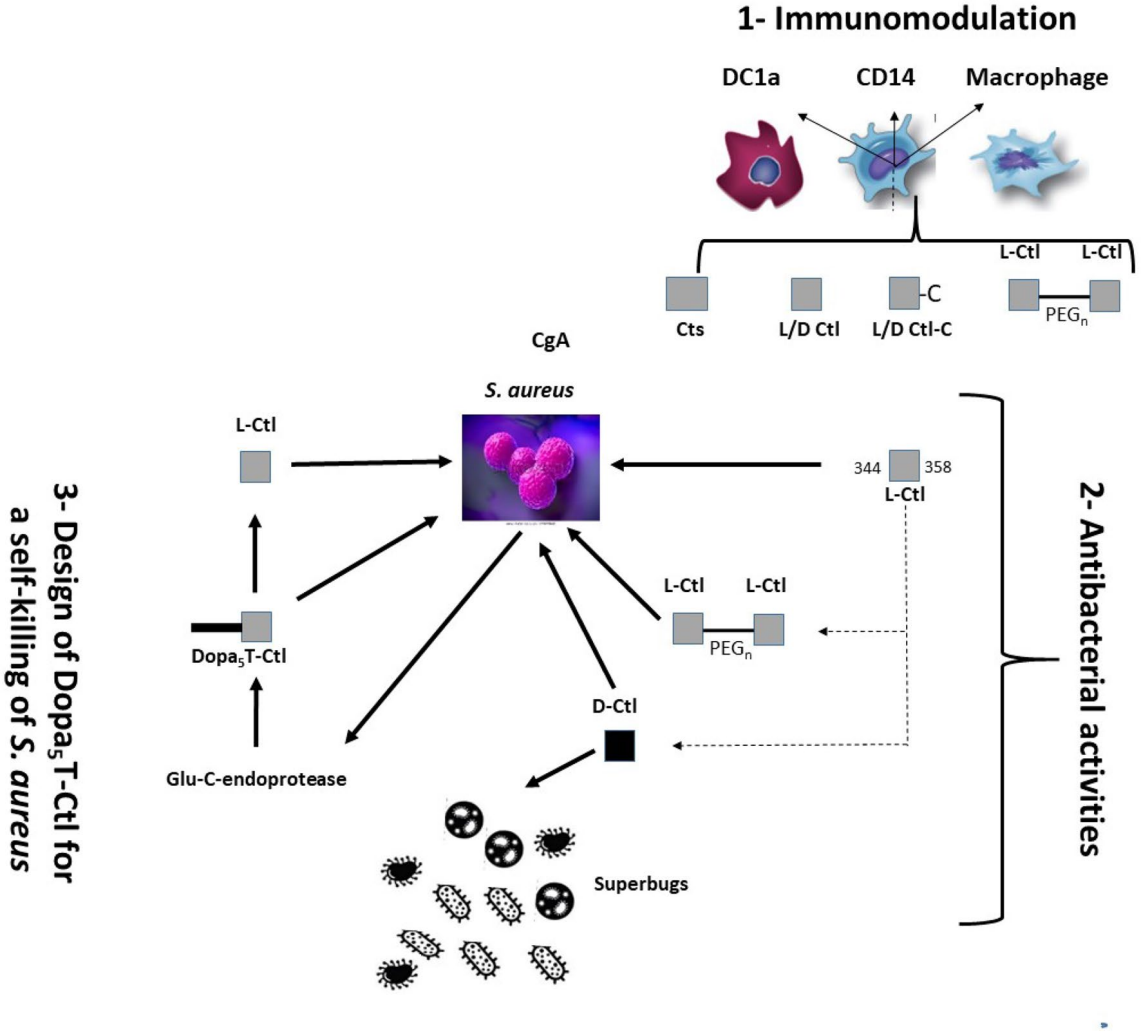
Outline of the study. 1—Immunomodulation: Interactions of Cts and its derived-peptides with immune cells are investigated; 2—The antibacterial activities of Ctl and the derived- peptides are examined; 3—The tri-blocks peptide Dopa_5_T-Ctl for the on-demand delivery of Ctl is tested.

This manuscript focuses on the ability of Cts-derived peptides at killing superbugs and more particularly *S. aureus*, a pathogen important in dermal infections^27^. The outline of the present study is (i) to investigate the interaction of Cts with dermal immune cells (dendritic cells DC1a, dermal macrophages CD14 and macrophages) and to analyze the cytokines release, (ii) to screen the antimicrobial activity of several Cts-derived peptides against superbugs (dimers separated by PEG linkers, D-isomer) and (iii) to design a new Ctl-derived peptide able to induce an on-demand self-killing of *S. aureus* (Fig. 1). This peptide corresponds to a tri-blocks peptide DOPA-K-DOPA-K-DOPA-TLRGGE-RSMRLSFRARGYGFR (DOPA_5_T-Ctl, Supplementary Fig. 1), which might release Ctl after proteolysis by the endoprotease Glu-C (E.C. 3.4.21.19) produced by *S. aureus*^28^. To design this peptide, functional molecules were attached using a previously reported technology^29,30^.

This study underscores that (i) the dimeric form of Ctl linked by 3 PEGs enhances the antibacterial activity against *S. aureus*, (ii) the D-Ctl peptide displays activity against superbugs, and (iii) the peptide DOPA_5_T-Ctl is not toxic and could be used in new strategies for the self-killing of *S. aureus* and the modulation of cellular immunity based on the localized and sustained release of Ctl.

## Results

### Interaction of Cts with immune dermal cells

**Figure 2:**
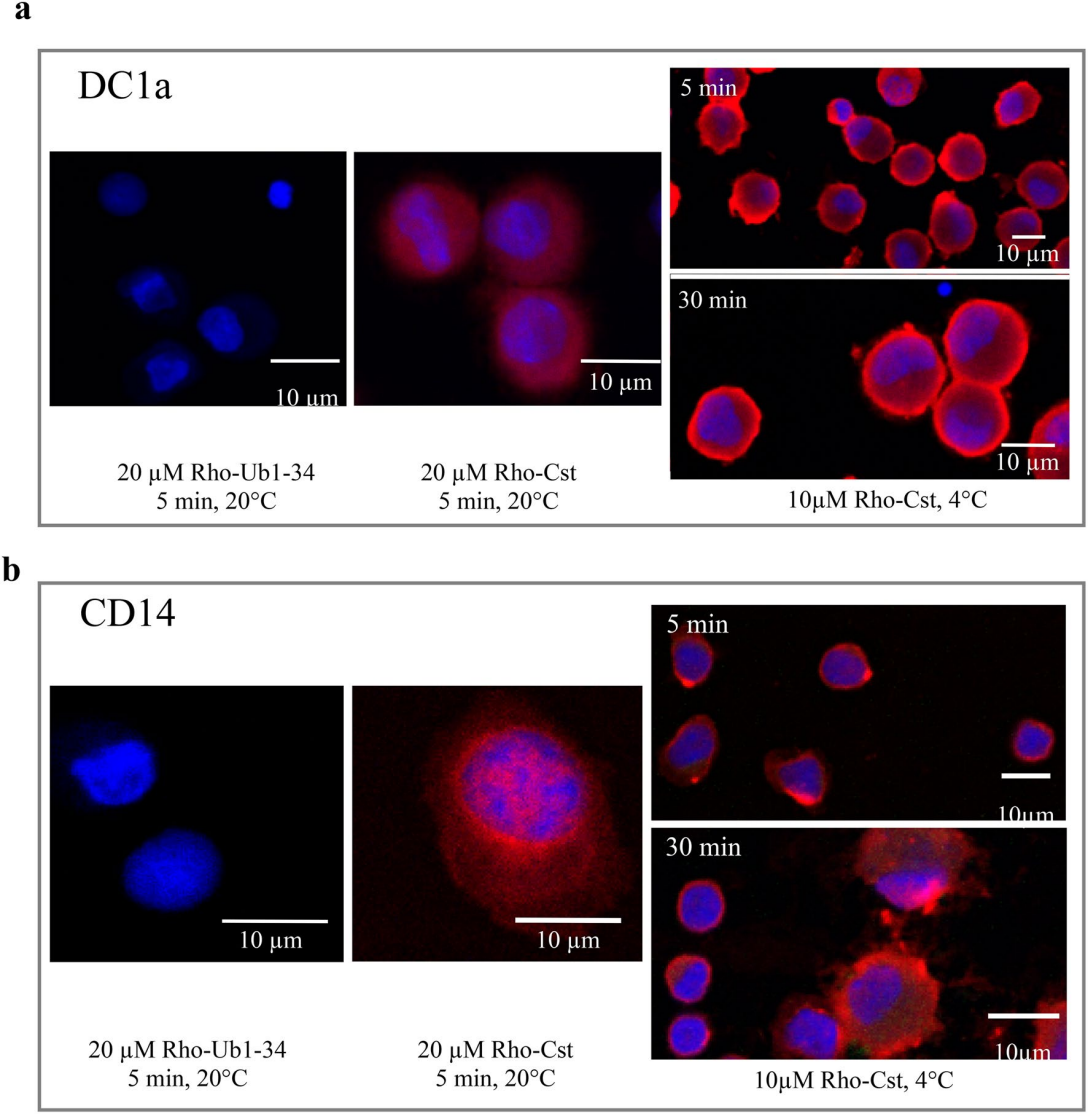
Confocal laser microscope observation of living DC1a (**a**) and CD14 (**b**) cells incubated with rhodamine labeled peptides: 20 µM Rho-Ub1-34 and 20 µM Rho-Cts during 5 min at 20 °C and 10 µM Rho-Cts during 5 min and 30 min at 4 °C.

**Figure 3:**
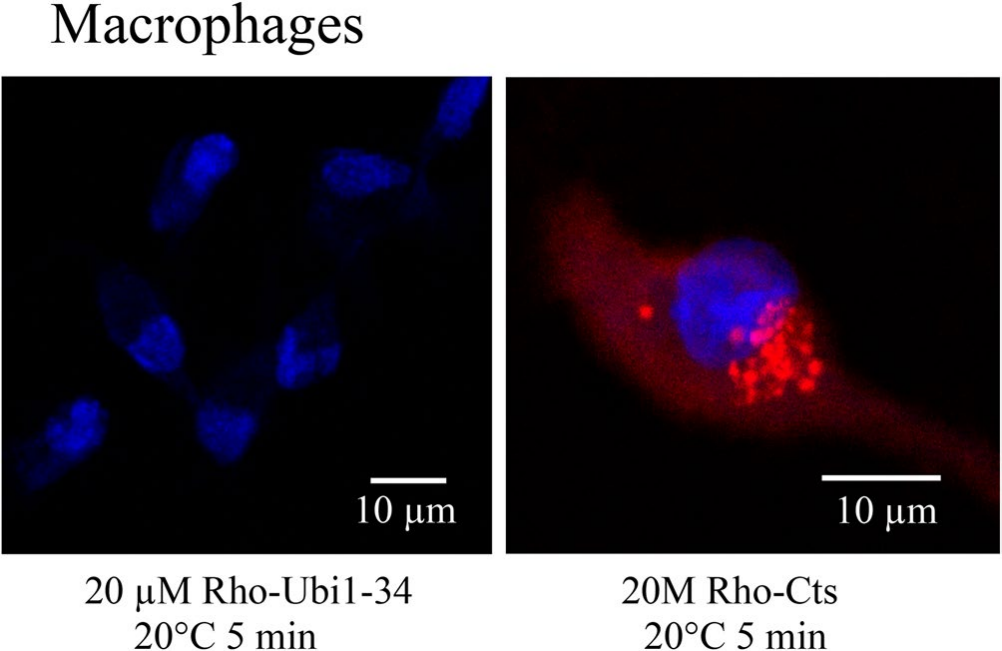
Confocal laser microscope observation of living macrophages incubated with rhodamine labeled peptides: 20 µM Rho-Ub1-34 and 20 µM Rho-Cts during 5 min at 20 °C.

The sequence of Cts is homologous to that of Penetratin, a CPP (Cell Penetrating Peptide), which penetrates into neutrophils^14^. Herein, we examined the interaction of Cts with different immune cells (Figs. 2 and 3) playing a role in dermal infection development (i.e.: dendritic cells DC1a, dermal macrophages CD14 and macrophages^31,32^).

Confocal laser microscope analysis of rhodaminated-Cts (Rho-Cts) incubated with cells was compared with that of Rho-Ub1-34, a peptide unable to penetrate into cells^33^. We detected the penetration and the subsequent localization of 20 µM Rho-Cts into the three types of immune cells. After 5 min of incubation with DC1a, 20 µM Rho-Cts was detected in the cytoplasm and nucleus (Fig. 2a), whereas its immune-localization was rather accumulating at the nuclear membrane and in some nuclear structures in CD14 cells (Fig. 2b). Finally, in macrophages, we observe a cytoplasmic localization of Rho-Cts with an accumulation in perinuclear vesicles (Fig. 3). In order to investigate the mechanism by which Rho-Cts is penetrating, experiments (performed at 4 °C for 5 and 30 min) confirmed that 20 µM Rho-Cts is able to penetrate into DC1a and CD14 indicating that endocytosis is not the mechanism of penetration (Fig. 2). These data confirmed that Cts is a CPP and suggest the same property for its active domain Ctl.

**Figure 4:**
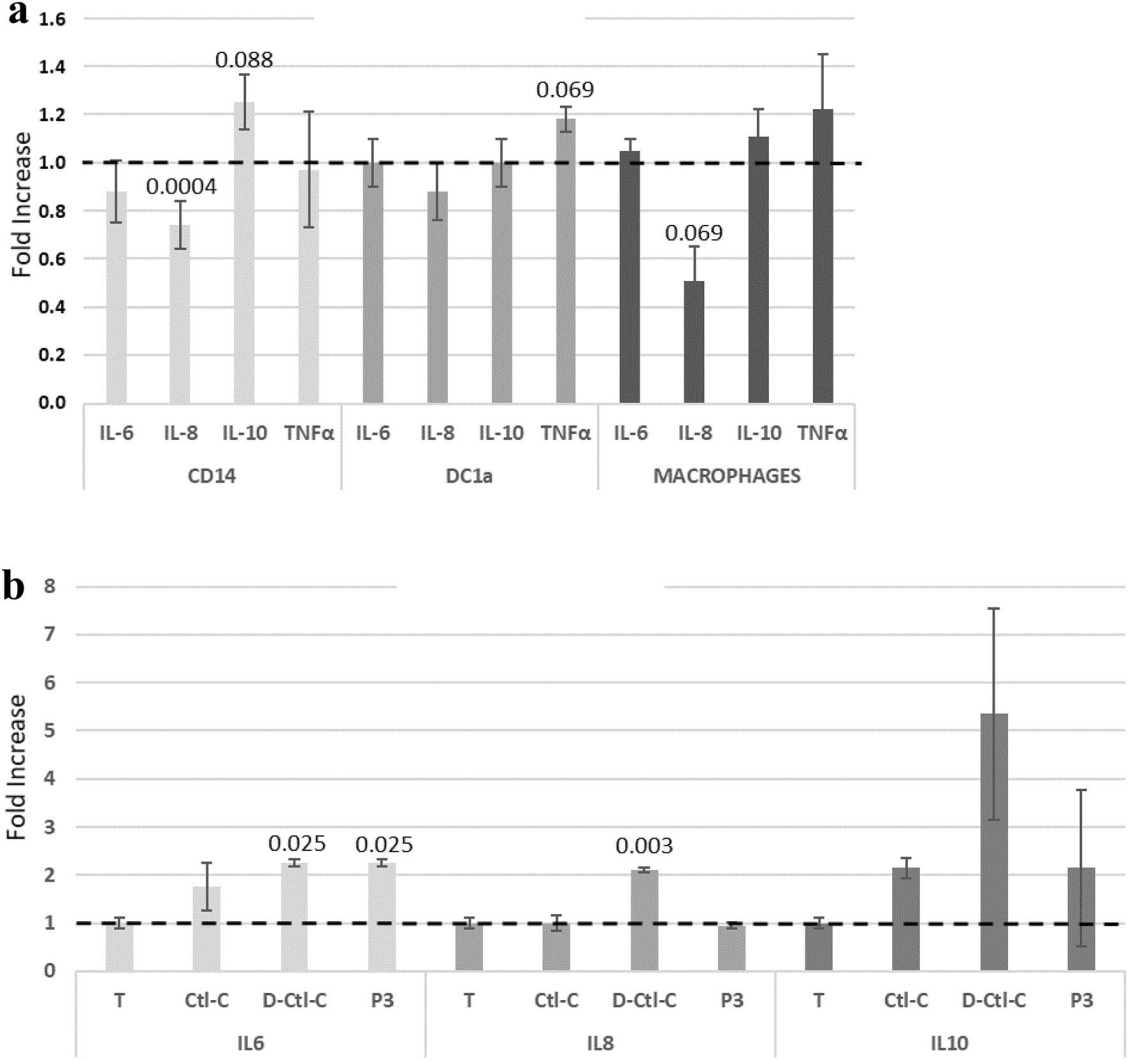
Cytokines assay release: (**a**) Cells (CD14, DC1a and macrophages) from two healthy volunteers were treated with 15 µM Cts from 24 h. The cytokines levels were evaluated in the cell supernatant using the ELISA assay. Fold increase represents the ratio of cytokine release induced by Cts/cytokine release control (without peptide). Data are shown as mean + /-standard deviation of two independent experiments; p are indicated. (**b**) PBMCs from two healthy volunteers were treated with the dimeric form P3 (3 PEGs), Ctl-C and D-Ctl-C. The cytokines levels were evaluated in the cell supernatant using the Bio-Plex technic. Fold increase represents the ratio of cytokine release induced by Cts/cytokine release control (without peptide). Data are shown as mean + /−standard deviation of two independent experiments; p are indicated.

In addition, we have examined the release of 4 cytokines, involved in the mechanisms of inflammation, after treatment of cells (harvested from 2 patients) with 15 µM Cts (Fig. 4a). IL-6, IL-8, TNF-α display pro-inflammatory properties and IL-10 anti-inflammatory properties^34^. For CD14, the comparison of the amount of IL-8 released after activation by Cts and controls (treated without peptide), shows a significant decrease of 26%. Furthermore, the production of IL-6, IL-10 and TNF-α is not modified after treatment with Cts.

In order to optimize the antimicrobial activities of Ctl, the active domain of Cts, several dimers separated by PEG linkers and D-isomer forms were synthesized and tested (Fig. 1).

### Assessment of the antimicrobial activities of dimeric forms of Ctl against *S. aureus* and *C. albicans*

Peptides covalent dimers with spacers of n PEG caused rapid and potent killing of pathogenic bacteria^35^. Four different peptides (P3-P6) corresponding to the dimeric form of Ctl with spacers of n PEG (n = 3, 12, 16, 46) were tested against methicillin sensitive *S. aureus* (MSSA, strain 49775), methicillin resistant *S. aureus* (MRSA, strain S1) and *C. albicans*, (ATCC^©^ 10231TM). In recent years, *Candida* isolates with acquired resistance to azoles and echinocandins have been reported more frequently^36^. The antimicrobial activities were compared with Ctl and Ctl-C (P1, P2) (Table 1). A cysteine residue and a maleimide group were added at the C-terminal end of each monomer used with 3, 12, 16 and 46 PEGs (P3–P6). The syntheses were based on the reaction of a linker ended on both sides by 2 maleimide groups and the thiol group of the Ctl-C through the thiolene click reaction. The dimer P3 with 3 PEGs was the only one active against MSSA and MRSA with a MIC of 30 µM and 50 µM respectively, and also against *C. albicans* with a MIC of 20 µM, (Table 1). To explain the specific activity of P3 we propose that the longer PEGs prevent the penetration of the peptide with their antiadhesive properties.

**Table 1:** Antimicrobial activity against *S. aureus* and *C. albicans* of dimeric forms of Ctl. MSSA (strain 49775) and MRSA (strain S1); *C. albicans,* (ATCC 10231TM). ND, not determined.

Peptide	Sequence	MIC_100_ (µM)		
		MSSA	MRSA	*C. albicans*
P1	RSMRLSFRARGYGFR	50	100	20
P2	RSMRLSFRARGYGFRC	60	100	20
P3	RSMRLSFRARGYGFRC-(3PEG)-CRFGYGRARFSLRMSR	30	50	20
P4	RSMRLSFRARGYGFR*Mal*-(12PEG)-*Mal*RFGYGRARFSLRMSR	> 100	ND	ND
P5	RSMRLSFRARGYGFR*Mal*-(16PEG)-*Mal*RFGYGRARFSLRMSR	> 100	> 100	50
P6	RSMRLSFRARGYGFR*Mal*-(46PEG)-*Mal*RFGYGRARFSLRMSR	> 100	ND	ND

We have examined the ability of 30 µM of each peptide (Ctl-C, D-Ctl-C and P3) to release cytokines from PBMCs by using the Bio-Plex Multiplex Immunoassay system (Bio-Rad) which assesses the release of 17 cytokines. We have pointed out the evalutions obtained for IL-6, IL-8, IL-10 and TNF-α (Fig. 4b). We confirmed the data previously obtained for L- and D- Ctl indicating that these 2 peptides and P3 are unable to induce te release of the tested cytokines. The high variation in TNF-α release does not allow to conclude and it is not presented.

### Antimicrobial activities of L- and D- isoforms of Ctl against 79 Gram negative resistant bacteria and *C. albicans*

D- peptides are resistant to enzyme degradation and are therefore potent antimicrobial agents against pathogenic bacteria^25,26,37^.The antimicrobial activities of the L- and D-isoforms of Ctl (Table 2) were compared with the corresponding scrambled sequences against 75 Gram negative resistant bacteria and against *C. albicans* isolates with acquired resistance to azoles and echinocandins^36^. In addition to wild type, some bacterial strains were noted ESBL, (Extended Spectrum Beta-Lactamase), the most influential mechanism for cephalosporin resistance^38^. Among these strains some are noted CTXM, (the highest level for cefotaxime resistance) a rapidly growing family of ESBLs with significant clinical impact^39^. We can point out that the L- and D-scrambled sequences are inactive against all the tested strains with MICs > 128 µg/mL (> 68.8 µM) and that the D- isoform is always more active than the L-Ctl (Table 2).

**Table 2:** Antimicrobial activities of L- and D- Ctl and the corresponding scrambled peptides (controls) against resistant pathogens (MIC, µg/mL).

Bacterial strain	Type		Reference	D-Ctl	Control D	L-Ctl	Control L
*Escherichia coli*	Wild type		ATCC 6 (25,922)	32	> 128	128	> 128
			Ec 4	16	128		
			Ec 204	32	128		
	BLSE	CTXM	Ec 46 (C11)	16	128	> 128	> 128
		CTXM	Ec 47 (C12)	16	> 128		
		CTXM	Ec 70	64	> 128		
			Ec 73	128	> 128	> 128	> 128
			Ec 74	16	128		
			Ec 195	8	64		
	OXA 48	BLSE	Ec 71	16	128		
			Ec 197	16	64		
			Ec 198	16	128		
*Klebsiella pneumoniae*	Wild type		B-24	32	> 128	> 128	> 128
			B-73 (C3)	32	128		
			B-75 (C4)	32	128		
	BLSE	CTXM	B-49 (C2)	32	128	128	> 128
			B-50	32	> 128		
		CTXM	B-68 (C1)	32	> 128		
	KPC		B-97	> 128	> 128		
		BLSE	B-101	32	> 128		
			B-102	128	> 128		
*Enterobacter cloacae*	Wild type		B-141	128	> 128	> 128	> 128
			B-142	32	128		
			B-144	64	> 128		
	BLSE		B-43	32	> 128	> 128	> 128
			B-57	32	128		
			B-167	32	> 128		
	Amp C		B-38	32	> 128		
			B-44	32	> 128		
			B-96	32	128		
	0XA 48	BLSE	B-112	64	128		
		BLSE	B-113	32	128		
		BLSE	B-118	32	128		
*Enterobacter aerogenes*	Wild type		B-145	32	128	128	> 128
			B-146	32	128		
			B-147	32	128		
	BLSE		B-168	32	> 128	> 128	> 128
			B-169	64	128		
			B-170	32	128		
	AMP C		B-148	32	128		
			B-149	32	128		
			B-150	32	128		
*Serratia marcescens*	Wild type		B-151	> 128	> 128	> 128	> 128
			B-152	> 128	> 128		
			B-153	128	> 128		
	Amp C		B-154	> 128	> 128	> 128	> 128
			B-155	128	> 128		
			B-156	128	> 128		
*Morganella morganii*	Wild type		B-157	> 128	> 128	> 128	> 128
			B-158	> 128	> 128		
			B-159	> 128	> 128		
	Amp C		B-59	> 128	> 128	> 128	> 128
			B-160	> 128	> 128		
			B-161	> 128	> 128		
Citrobacter freundii	Xild type		B-162	32	128	128	> 128
			B-163	32	128		
			B-164	32	128		
	Amp C		B-65	64	128	> 128	> 128
			B-165	32	128		
			B-166	64	128		
MSSA	Wild type		ATCC1 (29,213)	64	> 64	> 128	> 128
			SA 100	64	> 128		
			SA 112	32	> 128		
MRSA	Wild type		ATCC 21	64	> 128	> 128	> 128
			SA 111	64	> 128	> 128	> 128
			SA 166	64	> 128		
*Pseudomonas aeruginosa*	Wild type		P-122	> 128	> 128	> 128	> 128
			P-129	128	> 128		
			P-131	128	> 128		
	Amp C		P-124	128	> 128	> 128	> 128
			P-125	128	> 128		
			P-85	128	> 128		
	VIM		P-144	128	> 128		
			P-149	64	> 128		
			P-150	128	> 128		
*Candida albicans*	Wild type		L-1	256	512	512	512
			L-2	256	512		
			L-3	256	512		

For *E. coli*, D-Ctl is active at 8 µg/mL (4.3 µM) and 16 µg/mL (8.6 µM) against EC195 (Amp C strain) and EC4, Ec46, Ec47, Ec74, Ec71, Ec197, Ec198 respectively. For *K. pneumoniae*, D-Ctl is active at 32 µg/mL (17.2 µM) for B24, B73, B75, (wild type), B49, B50, B68 (CTXM) B101 (ESBL). For *E. cloacae*, a frequent bacteria isolated in human clinical sample, D-Ctl is active at 32 µg/mL (17.2 µM) for B142 (wild type), B43, B57, B167 (ESBL), B38, B44, B96 (Amp C), B113 and B118 (ESBL and OXA 48). Similar data were obtained for: (1) *E. aerogenes and C. freundii*, with a MIC for D-Ctl of 32 µM (17.2 µM) against the strains B145, B146, B147 (wild type), B168, B170 (ESBL), B148, B149, B150 (Amp C); (2) for *E. aerogenes* and B162, B163, B164 (wild type), B165 (Amp C); (3) for C. *freundii*. All these strains are frequently involved in the the urinary tract infections.

In addition, D-Ctl is active against SA 112 (MSSA) with a MIC of 32 µg/mL (17.2 µM), whereas it is active with a MIC of 64 µg/mL (34.4 µM) for the others strains. D-Ctl is less active against *P. aeruginosa* with a MIC varying from 64 µg/mL (34.4 µM) to 128 µg/mL (68.8 µM) and it is inactive against the highly resistant strains L1, L2 and L3 of *C. albicans*.

Because *S. aureus* is an opportunistic pathogen and the leading cause of a wide range of severe clinical infections^27^, we have designed a new Ctl-derived peptide that will be used to fight this pathogen for on-demand self-killing.

### Monitoring of the on-demand release of Ctl from a tri-blocks peptide to kill S. aureus

**Figure 5:**
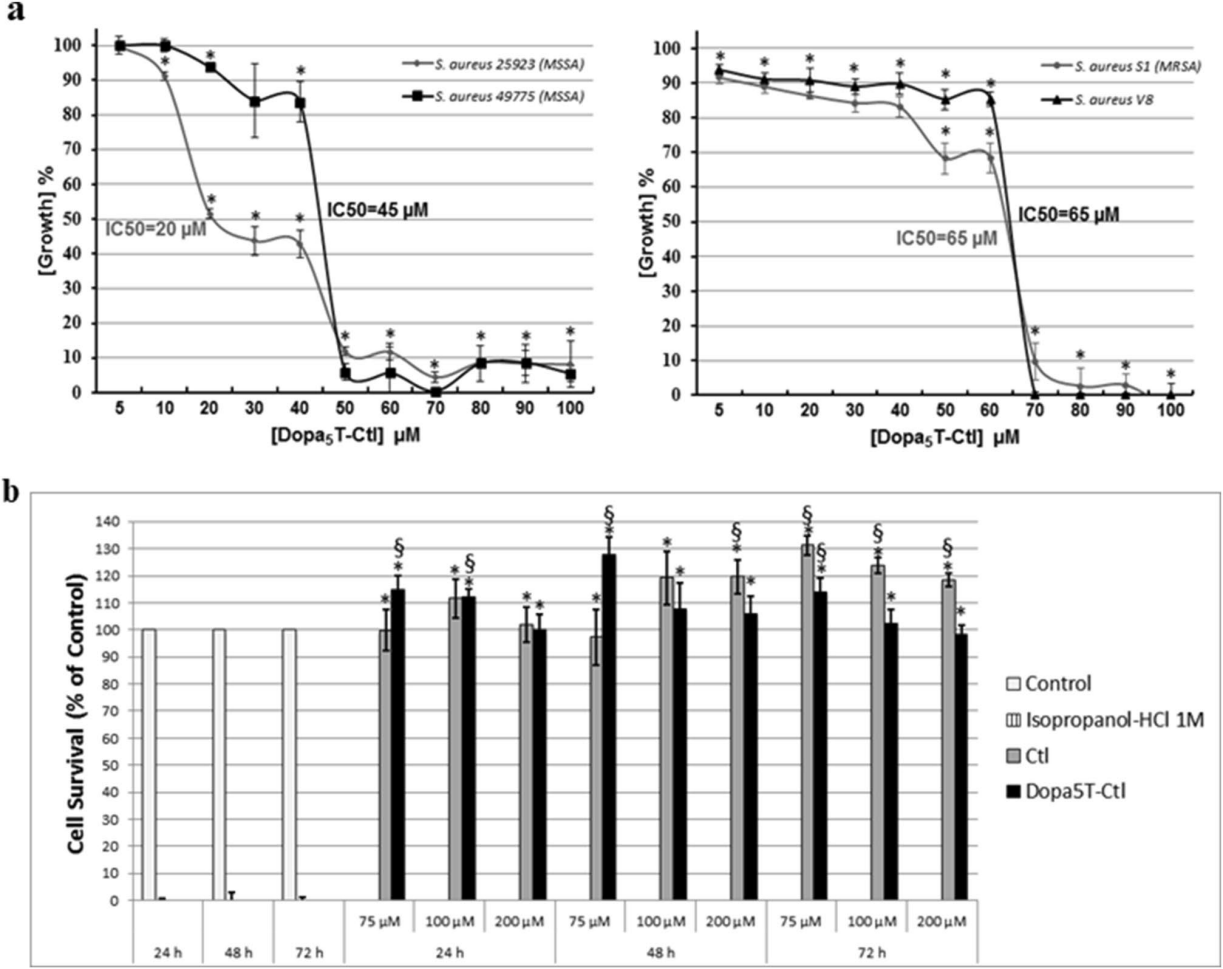
(**a**) Antimicrobial activity of Dopa_5_T-Ctl against different strains of *S. aureus*; t-test *p < 0.05 for comparison with the positive control, a mixture of Tetracycline (10 mg/L) and Cefotaxime (0.1 mg/L). Data are shown as mean + /−standard deviation of three independent experiments; (**b**) Toxicity assay against H9c2 cells. t-test §p < 0.05 for comparison with the positive control **(**100% of viability, vehicle**)** and *p < 0.05 for comparison with negative control (isopropanol-HCl 1 M). Data are shown as mean + /-standard deviation of three independent experiments.

The designed peptide DOPA_5_T-Ctl is a cationic peptide with a net charge + 7 (Supplementary Fig. 1). The three peptides Ctl, TCtl and DOPA_5_T-Ctl were characterized by RP-HPLC and Maldi-Tof mass spectrometry (Supplementary Fig. 2). DOPA_5_T-Ctl (5 to 100 µM) was tested against different strains of *S. aureus* including MSSA (strains 25923, 49775) and MRSA (strain S1 and V8) described in Methods (Fig. 5a)*.* The IC50 values of 20 µM, 45 µM, and 65 µM were respectively determined for the activity of DOPA_5_T-Ctl against MSSA (*S. aureus* 2523, *S. aureus* 49775) and MRSA (strain S1 and V8) (Fig. 4a). In similar experimental conditions, the antimicrobial properties of DOPA_5_T-(D)Ctl against *S. aureus* was investigated and the IC50 value of 35 µM was evaluated against the strain 49775 (Data not shown). Differences were all statistically significant (*p* < 0.05).

This antibacterial activity may result of the combination of a direct antimicrobial effect of DOPA_5_T-Ctl and the degradation of DOPA5T-Ctl by proteolytic enzymes from *S. aureus* and the consequent Ctl release*.* Indeed, the bacterial lysis induced by DOPA_5_T-Ctl provokes the release of proteolytic enzymes that may act to degrade DOPA_5_T-Ctl, but maintaining Ctl integrity as previously reported^22^. To investigate this hypothesis, we have tested the possibility of Ctl releasing from DOPA_5_T-Ctl after proteolysis by the Glu-C endoprotease (E.C. 3.4.21.19) produced by *S. aureus*^28^.

**Figure 6:**
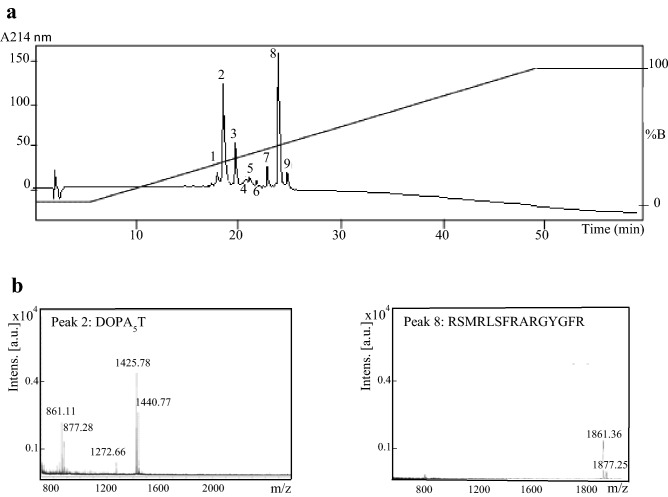
RP-HPLC and identification of the fragments obtained after incubation of DOPA_5_T-Ctl with the endoprotease Glu-C. (**a**) Chromatogram of the reverse –phase HPLC; gradient of elution is reported on the chromatogram (%B). (**b**) Maldi-Tof mass spectrometry of peaks 2 and 8 of the chromatogram. The Maldi-Tof mass spectrometry of minor peaks are reported in Supplementary Fig. 2.

DOPA_5_T-Ctl (1 mg) and T-Ctl (1 mg) were digested by Glu-C protease in a Tris–HCl solution according to the experimental procedure reported in Methods. The resulting peptides were isolated with RP-HPLC (Fig. 6a). Mass spectrometry of the major peaks 2 and 8, identified DOPA_5_T (1425.78 Da) and Ctl (1861.36 Da) with the corresponding oxidized forms (1440.77 Da and 1877.25 Da) (Fig. 6b and Table 3). This data demonstrates that the major fragments correspond to the cleavage of DOPA_5_T-Ctl after the glutamic residue (E) to induce the release of Ctl. Several products of degradation were also isolated: oxidized forms of DOPA_5_ (861.11 Da, 877.19 Da; Fraction 2) and DOPA_5_-TLRG (1272.66 Da; Fraction 2), (Fig. 6b and Table 3). Optical density measurement at 214 nm indicated the full proteolysis of DOPA_5_T-Ctl and 76% of Ctl and its oxidized form were released (Fig. 6a). The mass spectra of the minor fractions isolated after HPLC indicated that they correspond to the minor fragments previously indicated in Fractions 2 and 8, but also new fragments (Supplementary Fig. 3 and Table 3). Furthermore, we have shown that the DOPA_5_ sequence facilitates the release of the full peptide Ctl after action of the Glu-C endoprotease by comparison with T-Ctl which produces predominantly the fragments TLRGGE, RSMRLSFR and ARGYGFR (data not shown), with a non-specific cleavage site corresponding to the splitting of the peptidic bond R-A (Supplementary Fig. 1).

**Table 3:** Maldi-tof analysis of the 9 fractions obtained after HPLC of the digest of Dopa_5_T-Ctl by the endoprotease Glu-C.

Fraction	Molecular mass (Da)	Sequence
1	826.42	Dopa-K-Dopa-K-Dopa + 1(OX)
2	861.11	Dopa-K-Dopa-K-Dopa + 3(OX)
	877.10	Dopa-K-Dopa-K-Dopa + 4(OX)
	1272.66	Dopa-K-Dopa-K-Dopa-TLRG + 2(OX)
	1425.78	Dopa-K-Dopa-K-Dopa-TLRGGE
	1440.77	Dopa-K-Dopa-K-Dopa-TLRGGE + 1(OX)
3	861.31	Dopa-K-Dopa-K-Dopa + 3(OX)
	877.28	Dopa-K-Dopa-K-Dopa + 4(OX)
	893.26	Dopa-K-Dopa-K-Dopa + 5(OX)
	909.21	Dopa-K-Dopa-K-Dopa + -(OX)
	1069.75	RSMRLSFR + 1(OX)
	1193.98	TLRGGERSMR + 2(OX)
	1224.01	TLRGGERSMR + 4(OX)
	1295.04	RSMRLSFRAR + 1(OX)
	1426.03	Dopa-K-Dopa-K-Dopa-TLRGGE
	1449.99	Dopa-K-Dopa-K-Dopa-TLRGGE + 1(Na^+^)
4	1068.60	RSMRLSFR + 1(OX)
5	783.51	RSMRLS + 2(OX)
	920.50	ARGYGFR + 6(OX)
	1129.73	FRARGYGFR
6	1052.63	RSMRLSFR
	1068.62	RSMRLSFR + 1(OX)
7	1875.97	RSMRLSFRARGYGFR + 1(OX)
8	1861.36	RSMRLSFRARGYGFR
	1877.25	RSMRLSFRARGYGFR + 1(OX)
9	1633.07	MRLSFRARGYGFR + 1(OX)

### H9c2 cells viability

Ctl is not toxic for human gingival fibroblast (HGF-1)^26^ and human intestinal epithelial cell (caco-2)^25^. Herein, in relation with the properties of Ctl with the cardiac physiology, biocompatibility of DOPA_5_T-Ctl and Ctl was tested on Rat Cardiac Myoblast Cells (H9C2) with a MTT (3-(4,5-dimethylthiazol-2-yl)-2,5-diphenyl tetrazolium bromide) assay. Cell viability is defined as percentage of cells survival relative to that of positive controls (vehicle). For all the tested times of incubation (24, 48 and 72 h) and peptide concentrations (75, 100 and 200 µM) the cell viability was close or higher to 100% (*p* < 0.05) (Fig. 5b). DOPA_5_T-Ctl is not toxic for H9c2 cells. Similar data were obtained for Ctl (Fig. 5b) in line with the fact that the effect of L- and D-Ctl on H9c2 cells proliferation was recently reported indicating that L-Ctl (1–100 nM) induces a significant increase in cell viability in other settings^13^.

## Discussion

We report that Cts penetrates into DC1a, CD14 and macrophages (Figs. 2, 3) and its detection in the nucleus suggests a possible role in the transcription during neuro-immuno-cutaneous regulation. This data may be related with the fact that Cts penetrates also into PMNs^14^, activates human mast cells^15^ and induces monocytes chemotaxis^16^. It has also reported that Cts is upregulated upon injury^40^, thus demonstrating a direct link between the neuroendocrine and cutaneous immune systems.

The analysis of the release of 4 cytokines indicates the significant decrease of the pro-inflammatory cytokine IL-8 release from CD14 and suggests that Cts might inhibit the pro-inflammatory process. These data are in agreement with the property of Cts to modulate local intestinal inflammation through the shifting of macrophage polarization from the pro- to the anti-inflammatory phenotype^17,18^. The limitations of our study may be the low number of patients (2) and the role of Cts with transcription factors. However, according to previous data, Cts interacts with calmodulin to inhibit calcineurin^14^ and the action of Cts is mediated by the Ca^2+^-Calcineurin-NFAT signaling pathway^41^. Thus, the concept is emerging that Cts plays a role in tissue homeostasis by regulating immune cell infiltration and macrophage differentiation. In a previous paper it was reported that Cst may be incorporated into phospholipid membranes^42^ and that Ctl interacts with negative lipids inducing rigid membrane domains^43^.

In order to enhance the antimicrobial activity of Ctl against bacterial strains such as *S. aureus*, a series of modifications of the sequences were made on Cts-derived peptides, introducing cysteine, developing dimers separated by PEG linkers and on the D-isomer. Dimerization represents a potent strategy to develop novel antimicrobial agents efficient against resistant bacteria^44^. We have synthesized different peptides and compared their potency with that of the monomeric form Ctl: Ctl-C, Ctl-C-PEG3-C-Ctl and Ctl-Mal-PEG3-Mal-Ctl (n = 12, 16, 46).The structure–activity relationship of different dimeric forms of Ctl (the active domain of Cts) supports that the dimeric form including 3 PEGs (P3) improves the antibacterial activity against MSSA (50 µM; 93.0 µg/mL to 30 µM; 55.7 µg/mL) and MRSA (100 µM; 186 µg/mL to 50 µM; 93 µg/mL) (Table 1). By contrast, the activity against *C. albicans*, (a common pathogen of the skin), of the dimeric form P3 is similar to that of the monomeric molecule with a MIC of 20 µM; 37.3 µg/mL (Table 1).

In a new series of experiments we have examined the release of these cytokines after treatment of PBMCs with L- and D-Ctl, Ctl-C, D-Ctl-C and P3 (the short dimer) (Fig. 4b). We confirmed the data previously obtained for L- and D- Ctl and the dimeric form P3 is also unable to release significantly the tested cytokines. In contrast, the production of IL-8 is increased after activation with D-Ctl-C.

D- analogs of the HDPs were very stable against enzymatic proteolysis^25,26,35^ which suggests that their antibacterial activity may increase. This turned out to be always true for D-Ctl against several resistant bacteria (Table 2). The sequence of Ctl is of most importance for its activity because scrambled peptides did not inhibit microbial growth. D-Ctl is active against several strains of *E. coli* with a MIC of 8–32 µg/mL (4.3 µM-17.2 µM) which may be useful in infections such as that of the gut of newborns within hours after birth causing enterocolitis, urinary tract infections, meningitis and septicemia^45^. Indeed, this data is in accordance with previous results of our group showing that D-Ctl is a new HDP with undetectable susceptibility to resistance and potentiation of the efficiency of several antibiotics (cefotaxime, amoxicillin and methicillin)^25^. The D-isomer of Ctl is also active against *E. aerogenes and C. freundii* with a MIC of 32 µg/ml (17.2 µM). In this second part of the study it appears that the D-isomer of Ctl is the most potent antibacterial Ctl-derived peptide.

Recently, the activity of D-Ctl on two strains of *E. coli* has been examined by using biophysical technics. For all concentrations of D-Ctl, membrane permeabilization was shown, but no pore was observed^46^.

*S. aureus* is an important agent responsible for the majority of human skin and soft tissue infections^47^. We built up a non-toxic triblock peptide (DOPA_5_T-Ctl) for the on-demand self-killing of *S. aureus*. With a positive charge of + 7, DOPA_5_T-Ctl can facilitate the antimicrobial activities^4,5^. This study reports that DOPA_5_T-Ctl has the capacity to kill several strains of *S. aureus* (Fig. 5a) and that the Glu-C protease of *S. aureus* induces the production of the active Ctl (HPLC and Maldi-Tof mass spectrometry, Fig. 6a,b and Table 3). Antimicrobial activity of DOPA_5_T-(D)-Ctl is in the same range than that with (L)-Ctl with IC50 of 35 µM and 45 µM respectively. This data suggests that the D-isomer might prevent the action of the endoprotease Glu-C to release (D)-Ctl with a MIC of 17 µM.

The data obtained in this study show one additional link between the immune and neuroendocrine systems in which Cts interacts with dermal cells and may exert immunomodulatory effects on the cutaneous immune system. In addition, D-Ctl is the most potent Cts-derived antimicrobial peptide against superbugs and the design of a novel non-toxic molecule that permits localized on-demand delivery of an antimicrobial drug directly at the infectious site is described.

## Methods

### Preparation and characterization of synthetic Cts and the derived-peptides

The synthetic Cts and the Rhodamine labelled peptide were prepared as previously reported^9^. The chemically synthesized peptides corresponding to L- and D- isomer of Ctl (RSMRLSFRARGYGFR, purity > 95%) and the L and D isomer of the scramble sequence (FMRLRYRSSAFGGRR) were purchased from ProteoGenix (Schiltigheim, France). Dimeric forms of Ctl were synthesized at the Institute Charles Sadron UPR22 CNRS (Strasbourg, France) by Dr. Lydie Séon. These dimeric forms include spacers of n polyethylene glycol (PEG; n = 3, 12, 16 and 46); (PEG, 2000 Da, Iris Biotech, Marktredwitz, Germany). A cysteine residue and a maleimide group (*Mal*) is added at the end of each peptide before the binding to 3, 12, 16 and 46 PEGs (P3, P4, P5 and P6). TLRGGE-RSMRLSFRARGYGFR, (T-Ctl) and DOPA-K-DOPA-K-DOPA-TLRGGE-RSMRLSFRARGYGFR (DOPA_5_T-Ctl) were provided from Pepmic (Suzhou, China).

The purity of these peptides was tested by reverse phase (RP) HPLC^22^ with a Dionex HPLC system (Ultimate 3000; 13 Sunnyvale, USA) on a Vydac 208 TP C8 column (2.1 × 150 mm) equipped with a pre-column Vydac 208TP 14 (7.5 × 2.1 mm) (Vydac, AIT France, Houilles, France) (Supplementary Fig. 2). The solvent system consisted of 0.1% (vol/vol) trifluoroacetic acid (TFA) in water (solvent A) and 0.1% (vol/vol) TFA in 70% acetonitrile–water (solvent B) with a flow rate of 0.2 mL/min. Gradient of elution was indicated on chromatograms and each peak detected at λ214 nm was manually collected. Mass spectrometry measurements of material purified from each peak were determined using an Autoflex Maldi-Tof (Matrix Assisted Laser Desorption-Time Of Flight) mass spectrometer (Bruker Daltonics GmbH, Bremen, Germany) at the Laboratoire de Spectrométrie de Masse Bio-Organique, UMR7178 (CNRS-UDS, Strasbourg, France). The matrix solution was prepared from a saturated solution of α-cyano-4-hydroxycinnamic acid in water/acetonitrile 50/50 diluted three times in water/acetonitrile/trifluoroacetic acid 50/49.9/0.1^48^.

### Preparation of immune cells

Three types of immune cells were prepared from human monocytes provided from the EFS (Etablissement Français du Sang). Dendritic cells (DC1a), CD14 and macrophages were obtained after treatment of monocytes by cytokines according to the methods previously reported^31,32^ and the identification of the immune cells was validated by using flow cytometry analysis (Fluorescence Activated Cell Sorting, FACS).

### Treatment of immune cells by rhodaminated peptides

Rho-Cts (10 and 20 µM; 18.6 and 37.2 µg/mL) was incubated in Hepes buffer (140 mM NaCl, 5 mM KCl, 10 mM Hepes) with the three cell types (2.5 × 10^5^ for each assay) for 5 min at 20 °C. Then, the cells were washed three times with phosphate buffer saline (PBS). The cells were fixed with a solution of paraformaldehyde (PFA) 4% in PBS for 10 min and washed three times with PBS. Then, the nucleus were labelled for 5 min with DRAQ-5 (1, 5-bis{[2-(di-methylamino)ethyl]amino}-4, 8-dihydroxyanthracene-9, 10-dione; dilution 1/500) (BioStatus, Leicestershire, UK). A negative control was obtained with an inactive rhodamine labelled peptide 20 µM Rho-Ubi1-34, corresponding to the sequence Ubiquitin1-34^33^.

### Confocal microscopy

For immune cells treatment experiments, images were taken by confocal microscope. The acquisition was realized at the Plateforme Imagerie In vitro of Neuropôle (Strasbourg, France) by using a LSM 510 invert microscope Zeiss (Oberkofen, Germany) equipped with an immersion objective (× 63, N.A. 1.43).

### Antimicrobial assays against *S. aureus* and *C. albicans*

Antibacterial assays were realized to the method previously reported^22,25^. *S. aureus* strains were pre-cultured for 20 h in aerobic conditions at 37 °C in Mueller–Hinton Broth (MHB) medium (Becton–Dickinson Microbiology Company, Sparks, USA) (pH 7.4). Bacteria were suspended at absorbance of 0.001 at 620 nm in the MHB medium. Antibacterial activity was tested for 24 h incubation at 37 °C with shaking by measuring the inhibition of bacterial growth. Ten µl of final volumes (10–200 µg/mL) of synthetic peptides were incubated in microtitration plates with 90 µl of a mid-logarithmic phase culture of bacteria, with a starting absorbance of 0.001 at 620 nm. A mixture of Tetracycline (10 mg/L) and Cefotaxime (0.1 mg/L) were used as positive controls. Microbial growth was assessed by the increase of absorbance after 24 h incubation at 37 °C. The A620 nm value of control cultures growing in the absence of peptide and antibiotics was defined as 100% growth. A620 nm value with the antibiotics (Tetracycline and Cefotaxime) was taken as 100% inhibition. Absence of bacterial growth was verified by agar plate spreading. Each assay was performed in triplicates. Data were shown as mean + /− standard deviation of three independent experiments; p < 0.05.

Antimicrobial assays against *C. albicans* (ATCC 10231TM) were realized according to the method previously reported^26^. *C. albicans* was cultured in Sabouraud medium (Sigma-Aldrich, Saint Louis, USA) supplemented with tetracycline (10 μg/mL) and cefotaxime (10 μg/mL) at 37 °C for 24 h. *C. albicans* (OD620nm = 0.001) were plated in 96-well plates and treated either with different concentrations of the peptides of interest, and/or with different concentrations of voriconazole (VCZ) (Sigma-Aldrich, Saint Louis, USA). As a positive control, cells were treated with 10 μg/mL VCZ. After 24 h incubation, yeast growth was assessed by optical density OD620nm using a spectrophotometer Multiskan EX Microplate Reader Lab (Thermo Fisher Scientific, Waltham, MA, USA).

The MIC of the antifungal agents is defined as the lowest concentration of drug able to inhibit 100% of the growth of *C. albicans*.

### Antibacterial assays against resistant Gram-negative bacteria and *C. albicans*

The L- and D- forms of Ctl and also the corresponding L- and D-forms of the scramble peptides were tested by Atlangram (Nantes, France) against resistant Gram-negative bacteria (*Escherichia coli, Klebsiella pneumoniae*, *Enterobacter cloacae, Enterobacter aerogenes*, *Serratia marcescens, Morganella morganii, Pseudomonas aeruginosa),* MSSA, MRSA and yeast (*C. albicans)*. The MICs were determined according to the CASFM/EUCAST 2018 (Comité de l’Antibiogramme de la Société Française de Microbiologie).

### In vitro digestion of DOPA5T-Ctl by the Glu-C endoprotease

Endoprotease Glu-C from *S. aureus V8* (E.C. 3.4.21.19) was provided from Sigma Aldrich (Saint Louis, USA). Digestion of DOPA_5_T-Ctl (12.5 µg) by endoprotease Glu-C (Sigma-Aldrich Chemie Saint Quentin Fallavier, France) (3.5 µg) was obtained for 18 h at 37 °C in 50 mM Tris–HCl pH 8.2 or PBS^28^. Enzyme (1.75 µg) was added for a digestion of 4 h and then the same amount was added for 18 h. The enzymatic digestion was stopped by addition of 25 µL of 0.1% (vol/vol) TFA in milliQ water^49^. The fragments resulting from enzymatic digestion were isolated with a RP-HPLC with a Dionex HPLC system on a Vydac 208 TP C_8_ column (3 × 250 mm), and identified by Maldi-tof mass spectrometry, as previously reported for the characterization of the synthetic peptides^22^.

### Cells culture

H9c2 cells (rat embryonic cardiomyocytes) were obtained from American Type Culture Collection (ATCC, Manassas, VA, USA) (Cat# CRL-1446_H9c2, RRID:CVCL_0286) and maintained in Dulbecco’s Modified Eagle Medium/Nutrient Mixture F-12 (DMEM/F-12, Gibco, Thermo Fisher Scientific, Waltham, MA, USA) containing 10% fetal bovine serum (FBS, Gibco), supplemented with 1% penicillin/streptomycin (Thermo Fisher Scientific), and incubated at 37 °C in humidified atmosphere with 5% CO2. When the cells reached a density of 80% in 100-mm dishes, they were digested using 0.25% Trypsin–EDTA (1X) (Gibco) according to a 1:2 ratio following manufacturer’s instructions (ATCC)^50^.

### H9c2 cells viability

H9c2 cell viability was evaluated by 3-(4,5-dimethylthiazol-)2,5- diphenyl tetrazonium bromide (MTT) assay, as previously described^13^. Cells were seeded at a density of 5 × 103 cells/well in 96-well plate and incubated for 24 h, 48 h, 72 h in a 5% CO_2_ incubator at 37 °C to the following treatments: Positive Control (100% of viability, vehicle), negative control (1 M isopropanol-HCl), Ctl (75 µM, 100 µM, 200 µM) and Dopa5T-Ctl (75 µM, 100 µM, 200 µM). At the end of the treatments, the cell culture medium was replaced with 100 μl of 2 mg/ml MTT solution (Sigma Aldrich, Saint Louis USA) and cells were incubated for 3 h at 37 °C, 5% CO2. After incubation, the solution was removed and the formazan crystals were solubilized by adding 100 μl of DMSO for 30 min. The absorbance was measured using a Labsystems multiskan (RS-232C, Helsinki, Finland) at 570 nm. Multiskan EX Microplate Reader Lab (Thermo Fisher Scientific, Waltham, MA, USA) at 570 nm. The means of absorbance values of three wells in each experimental group were expressed as the percentage of cell viability. Cell viability was reported as percentage of cells survival relative to positive controls. Each assay was tested in triplicate and data are shown as mean + /− standard deviation of three independent experiments; p < 0.05.

### Cytokine release assays

The release of cytokines IL-6, IL-8, IL-10 and TNF-α induced after the treatment of immune cells by Cts was realized by the use of an automated ELISA immunoassay (Immulite One, Siemens la Garenne-Colombe, France) and with the Bio-Plex Multiplex Immunoassay system (Bio-Rad, Marnes-la-Coquette) for the treatment of Human Peripheral Blood Mononuclear Cells (PBMCs) by L-Ctl, D-Ctl, Ctl-C, D-Ctl-C and P3. PBMCs from healthy volunteers were obtained from the blood transfusion centre of Strasbourg (Etablissement Français du Sang, Strasbourg) and isolated by density gradient centrifugation using Lymphoprep (Stemcell Technologies). PMBC were then maintained in AIM V medium (Thermo Fisher Scientific, Waltham, MA, USA) at 37 °C in a 5% CO2 humidified incubator and treated for 24 h with 30 µM of each peptide (L-Ctl, D-Ctl, Ctl-C, D-Ctl-C and P3). Three technical replicates were performed for each condition. Supernatants were then filtered and assessed for cytokine dosage according to the manufacturer’s instructions.

## Supplementary Information

Supplementary Information.

## Data Availability

Sequence of Catestatin/Cateslytin: Uni protKB, CMGA_Bovin P05059.
