# Supplementary material for: Catestatin in innate immunity and Cateslytin-derived peptides against superbugs
Source: Sci Rep. 2021 Aug 2;11:15615. doi: 10.1038/s41598-021-94749-6 (PMC8329280; doi:10.1038/s41598-021-94749-6)
Supplement: Supplementary file 1 — Supplementary Information. [file 41598_2021_94749_MOESM1_ESM.docx]

Supplementary informations

**Catestatin in innate immunity and Cateslytin-derived peptides against superbugs**

Francesco Scavello^1,2^, Angela Mutschler^1^, Sophie Hellé^1,3^, Francis Schneider^1,3,4^,

Sylvette Chasserot-Golaz^5^, Jean-Marc Strub^6^, Sarah Cianferani^6^, Youssef Haikel^1,7^

and Marie-Hélène Metz-Boutigue^1#^

^1^, BioMaterials and BioEngeneering, Institut National de la Santé et de la Recherche Médicale UMR 1121, Federation of Translational Medicine Faculty of Odontology, University of Strasbourg, Strasbourg, France

^2^, Department of Biology, Ecology and Earth Science University of Calabria, Arcavacata di Rende, Italy

^3^, Faculty of Medicine, University of Strasbourg, Strasbourg, France

^4^, Medical Intensive Care, Hautepierre Hospital, Hôpitaux Universitaires, Strasbourg, Federation of Translational Medicine, Faculty of Medicine, University of Strasbourg, Strasbourg, France

^5^, Centre National de la Recherche Scientifique, Institut des Neurosciences Cellulaires et Intégratives, University of Strasbourg, Strasbourg, France

^6^, Centre National de la Recherche Scientifique, Laboratory of Bio-Organic Mass Spectrometry, Analytical Sciences Department, Pluridisciplinary Institute Hubert Curien, UMR 7178, University of Strasbourg, France

^7^, Faculty of Odontology, University of Strasbourg, Strasbourg, France.

# Corresponding author: Marie-Hélène Metz-Boutigue Institut National de la Santé et de la Recherche Médicale UMR_S 1121, Faculty of Odontology, Hôpital Civil, Porte de l’Hôpital 67000 Strasbourg, France.

Telefon: 0033368855471 ; Fax : 0033368855472 ; E-mail: [marie-helene.metz@inserm.fr](mailto:marie-helene.metz@inserm.fr).

Supplementary Figure 1


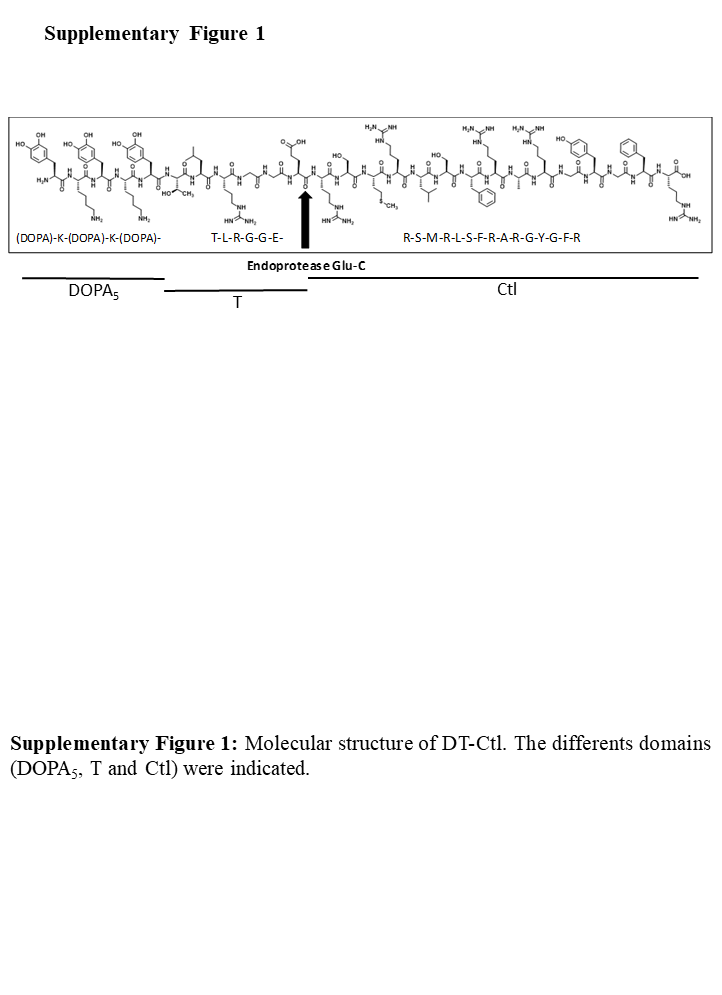


Supplementary Figure 2

**b**

**a**

**b**

Supplementary Figure 3

**Legends of supplementary Figures**

**Supplementary Figure 1:** Molecular structure of DOPA_5_T-Ctl. The different domains (DOPA_5_, T and Ctl) are indicated.

**Supplementary Figure 2:** Characterization of the peptides Ctl, T-Ctl and DOPA_5_T-Ctl. (a) The gradient of elution is reported on the chromatogram (%B). (b) The MALDI-TOF mass spectrometry of the isolated fractions is reported.

**Supplementary Figure 3:** MALDI-TOF mass spectrometry of the 9 peaks obtained after HPLC of the digestion of DOPA_5_T-Ctl with the endoprotease Glu-C.
